# Difficult Cases

**Published:** 1886-10-16

**Authors:** 


### Difficult Cases.

Mrs. Tebbutt, of Leyton, writes:—"In reply to the letter in the second nnmber of The Hospital, inquiring for a home for a lady with creeping paralysis, I beg to say I should be pleased to receive and take charge of her. Our house is large and private, with large garden attached, beautiful country views back and front. I am very fond of nursing, and possess considerable experience. She would receive every kindness, care, and attention. We have no children or lodgers, and have every accommodation for an invalid. The terms would be £2 per week. I can furnish medical and other references of the highest character.

*** We have also sent a letter from Miss Hawes to Mary H., and shall be glad if satisfactory arrangements result from this correspondence.—Ed. T. H.

